# Cu and Cu-SWCNT Nanoparticles’ Suspension in Pulsatile Casson Fluid Flow via Darcy–Forchheimer Porous Channel with Compliant Walls: A Prospective Model for Blood Flow in Stenosed Arteries

**DOI:** 10.3390/ijms22126494

**Published:** 2021-06-17

**Authors:** Amjad Ali, Zainab Bukhari, Muhammad Umar, Muhammad Ali Ismail, Zaheer Abbas

**Affiliations:** 1 Centre for Advanced Studies in Pure and Applied Mathematics, Bahauddin Zakariya University, Multan 60800, Pakistan; zainabbukhari@student.bzu.edu.pk (Z.B.); muhammadumar@bzu.edu.pk (M.U.); 2 Department of Computer and Information System Engineering, NED University of Engineering and Technology, Karachi 75270, Pakistan; maismail@neduet.edu.pk; 3 Department of Mathematics, The Islamia University of Bahawalpur, Bahawalpur 63100, Pakistan; zaheer.abbas@iub.edu.pk

**Keywords:** compliant walls, constricted channel, pulsatile flow, blood flow, nanoparticles, hybrid nanofluid

## Abstract

The use of experimental relations to approximate the efficient thermophysical properties of a nanofluid (NF) with Cu nanoparticles (NPs) and hybrid nanofluid (HNF) with Cu-SWCNT NPs and subsequently model the two-dimensional pulsatile Casson fluid flow under the impact of the magnetic field and thermal radiation is a novelty of the current study. Heat and mass transfer analysis of the pulsatile flow of non-Newtonian Casson HNF via a Darcy–Forchheimer porous channel with compliant walls is presented. Such a problem offers a prospective model to study the blood flow via stenosed arteries. A finite-difference flow solver is used to numerically solve the system obtained using the vorticity stream function formulation on the time-dependent governing equations. The behavior of Cu-based NF and Cu-SWCNT-based HNF on the wall shear stress (WSS), velocity, temperature, and concentration profiles are analyzed graphically. The influence of the Casson parameter, radiation parameter, Hartmann number, Darcy number, Soret number, Reynolds number, Strouhal number, and Peclet number on the flow profiles are analyzed. Furthermore, the influence of the flow parameters on the non-dimensional numbers such as the skin friction coefficient, Nusselt number, and Sherwood number is also discussed. These quantities escalate as the Reynolds number is enhanced and reduce by escalating the porosity parameter. The Peclet number shows a high impact on the microorganism’s density in a blood NF. The HNF has been shown to have superior thermal properties to the traditional one. These results could help in devising hydraulic treatments for blood flow in highly stenosed arteries, biomechanical system design, and industrial plants in which flow pulsation is essential.

## 1. Introduction

The study of blood flow through an artery that has stenosis plays an important role in understanding cardiovascular diseases. The stenosis created is due to the undesired growth of lumen inside the blood vessels, which results in a reduction of normal blood flow. In severe cases, the blockage of arteries may lead to stroke, heart attack, or other cardiovascular diseases. The human blood flow is frequently modeled as a non-Newtonian fluid. Moreover, the geometry of channels with compliant walls, i.e., having constrictions on the walls models, has been used to model arteries that have stenosis. Young [1] studied in detail the behavior of blood flow in arteries that have stenosis. He explained the effect of stenosis on blood flow, pressure distribution, and wall shearing stress distribution along with the stenosis, velocity, and flow separation region near the stenosis. The Casson model is a non-Newtonian liquid model that is used to simulate shearing thinning and stress. This model will be shown in terms of mathematical expression in Section 2. As a result of the unique characteristics, the Casson fluid is considered as a promising rheological fluid model for human blood. The rheological behavior of blood in microcirculation is mostly dictated by red blood cells. As a result of their peculiar features, they are responsible for blood’s non-Newtonian behavior. Casson fluid flow in a channel using the Brinkman model was investigated by Chaturani and Upadhya [2]. They concluded that increasing yield stress and decreasing the value of permeability reduces the flow rate. These results can be used to treat the blood clots in a coronary artery. The pulsating motion study has significant importance while analyzing the blood flow. Due to the cyclic nature of the pulse, the blood supply in arteries is intrinsically erratic and pulsating. Its analysis is challenging in both clinical and analytical contexts. Shit and Roy [3] analyzed the effects of magnetic field and heat on the pulsatile flow of blood in the arteries with stenosis. They concluded that the heat transfer rate has a direct relation toward the magnetic parameter. Elahi et al. [4] investigated the heat and mass transfer of non-Newtonian fluid flow in arteries with mild stenosis having permeable walls. Haghighi and Asl [5] presented the mathematical modeling of the pulsatile blood flow via overlapping constricted tapered vessels. Amjad et al. [6] investigated the influence of the magnetic field on the flow behavior of Casson fluid moving through a constricted channel using Darcy’s law for the steady and pulsatile flows. They deduced that the magnetic parameter could be used to control the flow separation region. Zainab et al. [7] expanded the study to analyze the heat transfer of Casson fluid flow in a constricted channel. They concluded that temperature profile has a direct relation toward the thermal radiation parameter and inverse relation toward the Prandtl number. Amjad et al. [8] studied the pulsatile flow of non-Newtonian micropolar fluid flow in a constricted channel, and Umar et al. [9] expanded the study to analyzed the heat transfer effects. They concluded that the Nusselt number is an increasing function of the Reynolds number and Prandtl number. Amjad et al. [10] examined the pulsatile flow behavior of micropolar-Casson fluid in a constricted channel as an exemplar problem of blood flow. They reported the direct relation of the Casson fluid parameter toward the wall shear stress. Some other relevant studies can also be seen in [11,12,13].

Heat transfer analysis has gained importance among researchers due to its vast applications in industries and biomedical engineering. Different kinds of fluids are used as heat carriers. The common fluids usually used as a heat/energy carrier to improve the product quality in industries are water, ethylene glycol, vegetable oil, paraffin oil, etc. [14]. The thermophysical properties of such base fluids are enhanced by the suspension of nanoparticles (NPs) of highly conductive solids. Common examples of nanoparticles (NPs) include those of copper (Cu), silver $\left(\mathrm{Ag}\right)$, copper oxide (CuO), aluminum oxide $\left({\mathrm{Al}}_{2}O_{3}\right)$, titanium oxide $\left({\mathrm{TiO}}_{2}\right)$, single-wall carbon nanotubes (SWCNTs), and multi-wall carbon nanotubes (MWCNTs). The literature reveals that remarkable efforts have been made by scientists to enhance heat transfer using different NFs. Recently, nanofluids (NFs) have been used in cancer therapeutics, hyperthermia, and drug carriers in biomedical engineering. The science of cardiovascular mechanics, in which an irregular plaque forms in the arterial lumen, as well as other fields such as biomedical engineering, chemical engineering, environmental degradation, and so on, benefit greatly from the dispersion of solute into vessels. The NFs formed using more than one type of NP are so-called hybrid nanofluids (HNFs). HNF might serve the desired purpose by combining the properties of its constituent materials. As a result of the synergistic impact, HNFs are found to exhibit better thermophysical characteristics compared with individual NFs [14]. Waini et al. [15] discussed the influence of transpiration on HNF flow and heat transfer for uniform shear flow over a stretching/shrinking surface. Lund et al. [16] analyzed the effects of different parameters on the flow profiles of an unsteady magnetohydrodynamic (MHD) flow of HNF mixture of copper–aluminum oxide/water over the stretching shrinking sheet in the existence of thermal radiation. Khan et al. [17] numerically studied impact variable viscosity in inclined MHD Williamson NF flow over a nonlinearly stretching sheet. They reported that the velocity profile declined with the rise of inclination angle, Hartmann number, and variable viscosity. The 3D flow of Cu-${\mathrm{AI}}_{2}O_{3}$/water HNF on an expanding surface was studied by Devi and Anjali [18] using the RK–Fehlberg integration technique. The numerical results demonstrated a higher heat transfer rate for the HNF than the copper-based NF. Vasua et al. [19] simulated the 2D rheological laminar hemodynamics via diseased tapered artery with mild stenosis theoretically and computationally. They considered the effect of different metallic NPs homogeneously suspended in the blood motivated by pharmacology applications. Priyadharshini and Ponalagusamy [20] investigated the blood flow having magnetic NPs in a stenosed artery. Some examples of relevant studies can also be found in [21,22,23,24].

Aman et al. [25,26] considered a novel HNF model with advanced thermophysical properties for an MHD Casson fluid in a porous vertical medium. They used alumina and copper NPs and analyzed the heat and mass transfer effects. Kasim et al. [27] considered the heat transfer effects over an unsteady stretching sheet. They used blood fluid-based copper and alumina NPs. Nadeem et al. [28] analyzed the impact of chemical reaction in a Casson NF flow. Jamshed and Aziz [29] carried out a study to analyze the entropy generation and heat transfer analysis of a Casson HNF under the impact of transverse magnetic field thermal radiation and Cattaneo-Christov heat flux model. Aside from that reported, the following recent studies, such as those by Souayeh et al. [30], Ullah et al. [31], and Aziz and Afify [32], can be referred to as additional knowledge correlated with the Casson NF flows. Dinarvand et al. [33] demonstrated steady laminar mixed convection incompressible viscous and electrically conducting HNF (CuO- Cu/blood) flow over the plane stagnation point over a horizontal porous stretching layer with an external magnetic field, taking into account the induced magnetic field effect, which can be used in biomedical fields, especially in drug delivery. Reddy [34] investigated the model for electro-MHD flow over a stagnation point flow of HNF with non-linear thermal radiation and non-uniform heat source/sink that is implemented to blood-based NFs for two different NPs. For bacterial development in the heart valve, Elelamy et al. [35] examined a mathematical model using numerical simulation. NPS was employed for antibacterial and antibody characteristics. They employed non-Newtonian fluid of Casson micropolar blood flow in the heart valve for 2D, since antibiotics are widely believed to be homogeneously dispersed via the blood. El Kot et al. [36] investigated the behavior of a gold–titanium oxide NPs combination suspended in blood as a base fluid in a damaged coronary artery. Their major purpose is to examine the shed light on the HNF flows via a vertical diseased artery in the presence of the catheter tube with heat transfer.

The current research adds into the literature the heat as well as mass transfer analysis of non-Newtonian Casson HNF flow with Cu and SWCNT as the two types of constituting nanoparticles influenced by the Lorentz force in channel with compliant walls and Darcian porous medium. The unsteady governing equations are transformed using the vorticity-stream function technique, and the resulting model is solved using a finite difference method (FDM). The objective is to examine the cumulative impact of the applied magnetic field and thermal radiation on the wall shear stress (WSS), velocity, temperature, and concentration profiles. The flow regulating parameters for the present study include the Hartmann number (the magnetic parameter), Strouhal number (the pulsation parameter), radiation parameter, Darcy’s number (the porosity parameter), Casson fluid parameter, Reynolds number, Soret number, and Peclet number (the heat or mass diffusion parameter). By definition, the Peclet number encompasses the effects of the Prandtl number for heat diffusion. Furthermore, the influence of the flow parameters on the non-dimensional numbers such as the skin friction coefficient, Nusselt number, and Sherwood number is also discussed. The results of the current study would provide insight to the relevant researchers and engineers to design concerning products. The non-Newtonian pulsatile nanofluid flows help in understanding the influence of various metallic NPs homogeneously suspended in the blood, which is driven by drug trafficking (pharmacology) applications. A thorough interpretation with the computations of particular significance to pharmacological NP-mediated rheological blood flow in stenosed arteries may be considered in future research.

The remaining parts of the article are organized in the following manner. The mathematical modeling and its transformation into a solvable form, specifically using the stream and vorticity functions, is presented in Section 2. Section 3 discusses the numerical scheme and validation. Section 4 discusses the findings and related discussions. Section 5 contains the concluding remarks.

## 2. Mathematical Model and Formulation

### 2.1. Problem Statement

A two-dimensional pulsatile flow of a non-Newtonian Casson HNF in a porous channel with symmetrical constrictions under the impact of a uniform magnetic field perpendicular to the fluid flow and thermal radiation is considered. Along with it, the chemical reaction is also considered. NF based on Cu NPs and HNF based on Cu-SWCNT NPs are two different kinds of NFs considered in this study to enhance the heat and mass transfer. Furthermore, thermal equilibrium among the base fluid and NPs and no-slip conditions are considered for the current study. The constriction on the channel walls is formed using the following expression

$$y_{1}\left(x\right)=\left\{\begin{array}{cc} \frac{h_{1}}{2}\left[1+\cos\left(\frac{\pi x}{x_{0}}\right)\right], & \left|x\right|\leq x_{0}\\ 0, & \left|x\right|>x_{0} \end{array}\right.$$

(1) $$y_{2}\left(x\right)=\left\{\begin{array}{cc} 1-\frac{h_{2}}{2}\left[1+\cos\left(\frac{\pi x}{x_{0}}\right)\right], & \left|x\right|\leq x_{0}\\ 1, & \left|x\right|>x_{0} \end{array}\right.$$

The mathematical model for the Casson fluid is given as [10]

(2) $$\tau_{ij}=\left\{\begin{array}{cc} 2\left(\mu_{\beta }+p_{y}/\sqrt{2\pi }\right)e_{ij} & \pi >\pi_{c}\\ 2\left(\mu_{\beta }+p_{y}/\sqrt{2\pi_{c}}\right)e_{ij} & \pi <\pi_{c} \end{array}\right.$$

### 2.2. Governing Equations

**Figure 1:**
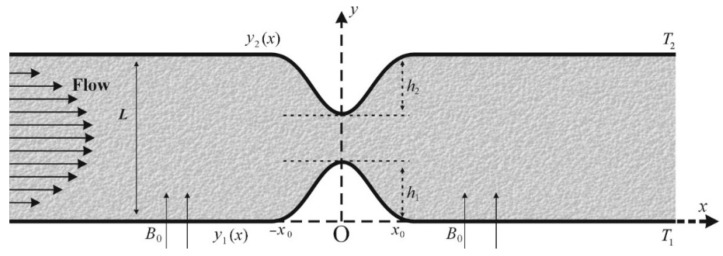
A schematic diagram of the flow channel with symmetrical constriction on both walls.

The walls of the channel have a pair of symmetric constriction bumps (see Figure 1). We suppose a Cartesian coordinate system $\left(\tilde{x},\tilde{y}\right)$ such that the flow direction is along the $\tilde{x}$-axis and the direction of B is along the $\tilde{y}$-axis. The resulting electric field J is normal to the plane of flow. In the transformed coordinate system (to be discussed later on), the constrictions are spanned from $x=-x_{0}$ to $x=x_{0}$ with its center at x=0, as shown in Figure 1. Thus, the length of the constriction is $2x_{0}$.

The flow phenomenon is represented by the unsteady incompressible viscous flow equations as follows.

The continuity equation:

(3) $$\frac{\partial \tilde{u}}{\partial \tilde{x}}+\frac{\partial \tilde{v}}{\partial \tilde{y}}=0$$

The momentum equation:

(4) $$\frac{\partial \tilde{u}}{\partial \tilde{t}}+\tilde{u}\frac{\partial \tilde{u}}{\partial \tilde{x}}+\tilde{v}\frac{\partial \tilde{u}}{\partial \tilde{y}}=-\frac{1}{\rho_{hnf}}\frac{\partial \tilde{p}}{\partial \tilde{x}}+\frac{\mu_{hnf}}{\rho_{hnf}}\left(1+\frac{1}{\beta }\right)\nabla^{2}\tilde{u}+\frac{1}{\rho_{hnf}}{\left(J\times B\right)}_{x}-\frac{\nu_{f}}{k}\tilde{u}$$

(5) $$\frac{\partial \tilde{v}}{\partial \tilde{t}}+\tilde{u}\frac{\partial \tilde{v}}{\partial \tilde{x}}+\tilde{v}\frac{\partial \tilde{v}}{\partial \tilde{y}}=-\frac{1}{\rho_{hnf}}\frac{\partial \tilde{p}}{\partial \tilde{y}}+\frac{\mu_{hnf}}{\rho_{hnf}}\left(1+\frac{1}{\beta }\right)\nabla^{2}\tilde{v}-\frac{\nu_{f}}{k}\tilde{v}$$

where $J\equiv \left(J_{x},J_{y},J_{z}\right)$, $B\equiv \left(0,B_{0},0\right)$.

The energy equation:

(6) $$\frac{\partial \tilde{T}}{\partial \tilde{t}}+\tilde{u}\frac{\partial \tilde{T}}{\partial \tilde{x}}+\tilde{v}\frac{\partial \tilde{T}}{\partial \tilde{y}}=\frac{k_{hnf}}{{\left(\rho C_{p}\right)}_{hnf}}\nabla^{2}\tilde{T}-\frac{1}{{\left(\rho C_{p}\right)}_{hnf}}\frac{\partial q}{\partial \tilde{y}}$$

where the radiative heat flux q defined as $q=-\left(\frac{4\sigma }{3k^{*}}4T_{\infty }^{3}\frac{\partial \tilde{T}}{\partial \tilde{y}}\right)$ and ${\tilde{T}}^{4}≅4T_{\infty }^{3}\tilde{T}-3T_{\infty }^{4}$ allows the expansion of $T^{4}$ using Taylor series about $T_{\infty }$ (free stream temperature); then, $q=-\left(\frac{4\sigma }{3k^{*}}4T_{\infty }^{3}\frac{\partial \tilde{T}}{\partial \tilde{y}}\right)$ so $\frac{\partial q}{\partial \tilde{y}}=-\left(\frac{16\sigma }{3k^{*}}T_{\infty }^{3}\frac{\partial^{2}\tilde{T}}{\partial {\tilde{y}}^{2}}\right)$. Equation (6) becomes

(7) $$\frac{\partial \tilde{T}}{\partial \tilde{t}}+\tilde{u}\frac{\partial \tilde{T}}{\partial \tilde{x}}+\tilde{v}\frac{\partial \tilde{T}}{\partial \tilde{y}}=\frac{k_{hnf}}{{\left(\rho C_{p}\right)}_{hnf}}\nabla^{2}\tilde{T}+\frac{16\sigma T_{\infty }^{3}}{3k^{*}{\left(\rho C_{p}\right)}_{hnf}}\frac{\partial^{2}\tilde{T}}{\partial {\tilde{y}}^{2}}$$

The concentration equation:

(8) $$\frac{\partial \tilde{C}}{\partial \tilde{t}}+\tilde{u}\frac{\partial \tilde{C}}{\partial \tilde{x}}+\tilde{v}\frac{\partial \tilde{C}}{\partial \tilde{y}}=D\nabla^{2}\tilde{C}+\frac{DK_{T}}{T_{m}}\nabla^{2}\tilde{T}$$

The expression J×B in Equation (4) is simplified using Ohm’s law and Maxwell’s equation. By Ohm’s law

(9) $$\begin{array}{ccc} J_{x}=0, & J_{y}=0, & J_{z}=\sigma \left(E_{z}+\tilde{u}B_{0}\right) \end{array}$$

Maxwell’s equation ∇×E=0 for steady flow implies that $E_{z}=a$, where a is a constant number. We can assume a to be zero. Then, Equation (9) gives, $J_{z}=\sigma \tilde{u}B_{0}$. Therefore, $J\times B=-\sigma \tilde{u}B_{0}^{2}$. So, Equation (3) becomes

(10) $$\frac{\partial \tilde{u}}{\partial \tilde{t}}+\tilde{u}\frac{\partial \tilde{u}}{\partial \tilde{x}}+\tilde{v}\frac{\partial \tilde{u}}{\partial \tilde{y}}=-\frac{1}{\rho_{hnf}}\frac{\partial \tilde{p}}{\partial \tilde{x}}+\frac{\mu_{hnf}}{\rho_{hnf}}\left(1+\frac{1}{\beta }\right)\nabla^{2}\tilde{u}-\frac{1}{\rho_{hnf}}\sigma_{f}\tilde{u}B_{0}^{2}-\frac{\nu_{f}}{k}\tilde{u}$$

The following dimensionless variables are considered to convert the governing Equations (3), (5), (7), (8) and (10) into the dimensionless form:

(11) $$\begin{array}{cccccccc} x=\frac{\tilde{x}}{L}, & y=\frac{\tilde{y}}{L}, & u=\frac{\tilde{u}}{U}, & v=\frac{\tilde{v}}{U}, & t=\frac{\tilde{t}}{T}, & St=\frac{L}{UT}, & p=\frac{\tilde{p}}{\rho_{f}U^{2}}, & Re=\frac{\rho_{f}UL}{\mu_{f}}, \end{array}\;\begin{array}{cccccc} M=B_{0}L\sqrt{\frac{\sigma_{f}}{\rho_{f}\nu_{f}}}, & D_{a}=\frac{\nu_{f}}{U\sqrt{k}}, & Pr=\frac{\mu_{f}C_{p,f}}{k_{f}}, & Rd=\frac{16\sigma T_{\infty }^{3}}{3k^{*}k_{f}}, & \varphi =\frac{\tilde{C}-C_{2}}{C_{1}-C_{2}}, & \theta =\frac{\tilde{T}-T_{2}}{T_{1}-T_{2}}, \end{array}\;\begin{array}{cccc} Sc=\frac{\mu_{f}}{\rho_{f}D}, & Sr=\frac{\rho_{f}DK_{T}\left(T_{1}-T_{2}\right)}{\mu_{f}T_{m}\left(C_{1}-C_{2}\right)}, & Peh=Pr\cdot Re, & Pem=Sc\cdot Re \end{array}$$

Here, *D* is the coefficient of mass diffusivity, $K_{T}$ is the thermal diffusion ratio, and $T_{m}$ is the mean temperature. The effective thermophysical properties of HNF [16,37] are presented as follows.

The HNF’s effective dynamic viscosity $\mu_{hnf}$ is given as

(12) $$\mu_{hnf}=\frac{\mu_{f}}{{\left(1-\phi_{1}\right)}^{2.5}{\left(1-\phi_{2}\right)}^{2.5}}$$

Here,$\;\phi_{1}$ and $\phi_{2}$ show the volume fractions of NPs Cu and SWCNT, respectively. The NF’s effective density $\rho_{hnf}$ is given as

(13) $$\rho_{hnf}=\left(1-\phi_{2}\right)\left[\left(1-\phi_{1}\right)\rho_{f}+\phi_{1}\rho_{s_{1}}\right]+\phi_{2}\rho_{s_{2}}$$

Here, $s_{1}$ and $s_{2}$ in subscripts correspond to the property of Cu and SWCNT, respectively. Moreover, hnf, nf, and f in the subscripts correspond to the property of the HNF, NF, and base fluid water, respectively.

The NF’s heat capacitance ${\left(\rho C_{p}\right)}_{hnf}$ is given as

(14) $${\left(\rho C_{p}\right)}_{hnf}=\left(1-\phi_{2}\right)\left[\left(1-\phi_{1}\right){\left(\rho C_{p}\right)}_{f}+\phi_{1}{\left(\rho C_{p}\right)}_{s_{1}}\right]+\phi_{2}{\left(\rho C_{p}\right)}_{s_{2}}.$$

The NF’s effective thermal conductivity $k_{hnf}$ is given as

(15) $$\frac{k_{hnf}}{k_{nf}}=\frac{k_{s_{2}}+2k_{nf}-2\phi_{2}\left(k_{nf}-k_{s_{2}}\right)}{k_{s_{2}}+2k_{nf}+2\phi_{2}\left(k_{nf}-k_{s_{2}}\right)}$$

where

(16) $$\frac{k_{nf}}{k_{f}}=\frac{k_{s_{1}}+2k_{f}-2\phi_{1}\left(k_{f}-k_{s_{1}}\right)}{k_{s_{1}}+2k_{f}+2\phi_{1}\left(k_{f}-k_{s_{1}}\right)}$$

where ϕ shows the volume fraction of NF, and the subscripts f and s correspond to the fluid and solid-state properties, respectively. For the present work, the nanoparticles of the following materials are considered: Cu and SWCNT.

The thermophysical properties of the base fluid water and the NPs under consideration are shown in Table 1. The properties in Table 1 are given at 24.6 °C. The viscosity of water at 24.6 °C is approximately 0.0000009009 m^2^/s. Thus, according to Equation (12), $\mu_{hnf}=7.940348428\times 10^{-7}$ m^2^/s with $\phi_{1}=\phi_{2}=0.03$.

Equations (3), (5), (7), (8) and (10) after transformation become

(17) $$\frac{\partial u}{\partial x}+\frac{\partial u}{\partial y}=0$$

(18) $$St\frac{\partial u}{\partial t}+u\frac{\partial u}{\partial x}+v\frac{\partial u}{\partial y}=-\frac{1}{\emptyset_{1}}\frac{\partial p}{\partial x}+\frac{1}{Re\emptyset_{3}}\left(1+\frac{1}{\beta }\right)\nabla^{2}u-\frac{1}{\emptyset_{1}}\frac{M^{2}}{Re}u-ReD_{a}^{2}u$$

(19) $$St\frac{\partial v}{\partial t}+u\frac{\partial v}{\partial x}+v\frac{\partial v}{\partial y}=-\frac{1}{\emptyset_{1}}\frac{\partial p}{\partial y}+\frac{1}{Re\emptyset_{3}}\left(1+\frac{1}{\beta }\right)\nabla^{2}v-ReD_{a}^{2}v$$

(20) $$St\frac{\partial \theta }{\partial t}+u\frac{\partial \theta }{\partial x}+v\frac{\partial \theta }{\partial y}=\frac{1}{Peh}\frac{\emptyset_{5}}{\emptyset_{4}}\left(\frac{\partial^{2}\theta }{\partial x^{2}}+\left(1+\frac{Rd}{\emptyset_{5}}\right)\frac{\partial^{2}\theta }{\partial y^{2}}\right)$$

(21) $$St\frac{\partial \varphi }{\partial t}+u\frac{\partial \varphi }{\partial x}+v\frac{\partial \varphi }{\partial y}=\frac{1}{Pem}\left(\frac{\partial^{2}\varphi }{\partial x^{2}}+\frac{\partial^{2}\varphi }{\partial y^{2}}\right)+\frac{Sr}{Re}\left(\frac{\partial^{2}\theta }{\partial x^{2}}+\frac{\partial^{2}\theta }{\partial y^{2}}\right)$$

where

$$\emptyset_{1}=\left(1-\phi_{2}\right)\left[\left(1-\phi_{1}\right)+\phi_{1}\frac{\rho_{s_{1}}}{\rho_{f}}\right]+\phi_{2}\frac{\rho_{s_{2}}}{\rho_{f}}$$

$$\emptyset_{2}=\frac{1}{{\left(1-\phi_{1}\right)}^{2.5}{\left(1-\phi_{2}\right)}^{2.5}}$$

$$\emptyset_{3}={\left(1-\phi_{1}\right)}^{2.5}{\left(1-\phi_{2}\right)}^{2.5}\left(\left(1-\phi_{2}\right)\left[\left(1-\phi_{1}\right)+\phi_{1}\frac{\rho_{s_{1}}}{\rho_{f}}\right]+\phi_{2}\frac{\rho_{s_{2}}}{\rho_{f}}\right)$$

$$\emptyset_{4}=\left(1-\phi_{2}\right)\left[\left(1-\phi_{1}\right)+\phi_{1}\frac{{\left(\rho C_{p}\right)}_{s_{1}}}{{\left(\rho C_{p}\right)}_{f}}\right]+\phi_{2}\frac{{\left(\rho C_{p}\right)}_{s_{2}}}{{\left(\rho C_{p}\right)}_{f}}$$

$$\emptyset_{5}=\frac{k_{hnf}}{k_{f}}$$

### 2.3. Stream and Vorticity Functions

The following transformation is used to transform the governing equations into the stream $\left(\psi \right)$ and vorticity $\left(\omega \right)$ functions:

(22) $$\begin{array}{ccc} u=\frac{\partial \psi }{\partial y}, & v=-\frac{\partial \psi }{\partial x}, & \omega =\frac{\partial v}{\partial x}-\frac{\partial u}{\partial y} \end{array}$$

The WSS $\left(\tau_{w}\right)$ is calculated using the vorticity at the wall, as both are orthogonal to each other [40]. Applying Equation (22) to Equations (18) and (19), and rearranging result:

(23) $$St\frac{\partial }{\partial t}\left(\frac{\partial v}{\partial x}-\frac{\partial u}{\partial y}\right)+u\frac{\partial }{\partial x}\left(\frac{\partial v}{\partial x}-\frac{\partial u}{\partial y}\right)+v\frac{\partial }{\partial y}\left(\frac{\partial v}{\partial x}-\frac{\partial u}{\partial y}\right)\;=\frac{1}{Re\emptyset_{3}}\left(1+\frac{1}{\beta }\right)\left[\frac{\partial^{2}}{\partial x^{2}}\left(\frac{\partial v}{\partial x}-\frac{\partial u}{\partial y}\right)+\frac{\partial^{2}}{\partial y^{2}}\left(\frac{\partial v}{\partial x}-\frac{\partial u}{\partial y}\right)\right]+\frac{1}{\emptyset_{1}}\frac{M^{2}}{Re}\frac{\partial u}{\partial y}+ReD_{a}^{2}\omega$$

(24) $$St\frac{\partial \omega }{\partial t}+\frac{\partial \psi }{\partial y}\frac{\partial \omega }{\partial x}-\frac{\partial \psi }{\partial x}\frac{\partial \omega }{\partial y}=\frac{1}{\emptyset_{3}}\frac{1}{Re}\left(1+\frac{1}{\beta }\right)\left[\frac{\partial^{2}\omega }{\partial x^{2}}+\frac{\partial^{2}\omega }{\partial y^{2}}\right]+\frac{1}{\emptyset_{1}}\frac{M^{2}}{Re}\frac{\partial^{2}\psi }{\partial y^{2}}+ReD_{a}^{2}\omega$$

The Equation for the stream function ψ (Poisson equation) is given by

(25) $$\frac{\partial^{2}\psi }{\partial x^{2}}+\frac{\partial^{2}\psi }{\partial y^{2}}=-\omega$$

Here u, v, θ, and φ are the primitive variables, whereas ω and ψ are the non-primitive variables.

### 2.4. Boundary Conditions

The Equation of motion (4) for the steady case, in the presence of electric and magnetic fields, becomes

(26) $$\mu_{hnf}\left(1+\frac{1}{\beta }\right)\frac{\partial^{2}\tilde{u}}{\partial {\tilde{y}}^{2}}-\sigma_{f}\tilde{u}B_{0}^{2}-\rho_{hnf}\frac{\nu_{f}}{k}\tilde{u}=\frac{\partial \tilde{p}}{\partial \tilde{x}}+\sigma_{f}B_{0}E_{z}$$

Again, for the steady flow, Maxwell’s equation, ∇×E=0 gives $E_{z}=a$, where a is a constant number, and all the other variables depend only on $\tilde{y}$, except for the pressure gradient, i.e., $\frac{\partial \tilde{p}}{\partial \tilde{x}}$ = constant.

Using the thermophysical properties and Equation (11), Equation (26) becomes

(27) $$\emptyset_{2}\left(1+\frac{1}{\beta }\right)\frac{d^{2}u}{dy^{2}}-\left(M^{2}+\emptyset_{1}Re^{2}D_{a}^{2}\right)u=\frac{L^{2}}{\rho_{f}\nu_{f}U}\left(\frac{\partial \tilde{p}}{\partial \tilde{x}}+\sigma_{f}B_{0}E_{z}\right)$$

For M≠0,

(28) $$\psi =\left[\frac{M^{2}\sqrt{g}\cosh\left(\frac{M}{2}\right)\left(\frac{M_{1}}{\sqrt{g}}\cosh\left(\frac{M_{1}}{2\sqrt{g}}\right)\eta -\sinh\left(\frac{M_{1}}{\sqrt{g}}\left(\eta -\frac{1}{2}\right)\right)\right)}{8M_{1}^{3}\sinh^{2}\left(\frac{M}{4}\right)\cosh\left(\frac{M_{1}}{2\sqrt{g}}\right)}\right]\times \left[1+\epsilon \sin\left(2\pi t\right)\right]$$

For M=0 and $D_{a}=0$,

(29) $$\psi =\frac{1}{6g}\left(3\eta^{2}-2\eta^{3}\right)\left[1+\epsilon \sin\left(2\pi t\right)\right]$$

For M=0 and $D_{a}\neq 0$,

(30) $$\psi =\frac{2}{Re^{2}D_{a}^{2}}\left[\eta -\frac{\sqrt{g}}{ReD_{a}}\left(\sinh\left(g_{1}\right)-\cosh\left(g_{1}\right)\tanh\left(\frac{g_{1}}{2}\right)\right)\right]\times \left[1+\epsilon \sin\left(2\pi t\right)\right]$$

Here,

$$\begin{array}{ccc} g=\emptyset_{2}\left(1+\frac{1}{\beta }\right), & M_{1}^{2}=\left(M^{2}+\emptyset_{1}Re^{2}D_{a}^{2}\right), & g_{1}=\frac{\sqrt{\emptyset_{1}}ReD_{a}\eta }{\sqrt{g}} \end{array}$$

where ϵ denotes the pulsating amplitude, ϵ=0 indicates the steady flow conditions, and ϵ=1 indicates the pulsatile flow conditions. For the temperature, the boundary conditions after transformation are given by, θ=1, at η=0; θ=0, at η=1. By substituting η=0 and η=1 in (28)–(30), the wall boundary conditions for ψ at the upper and lower walls are obtained.

The initial and boundary conditions for the variables (stream function ψ, the vorticity ω, temperature θ, and concentration φ) are given in the new coordinate system:

$$\begin{array}{cc} \psi =\omega =\theta =\varphi =0, & \mathrm{for}\;\mathrm{all}\;t\leq 0 \end{array}$$

The wall boundary conditions for ω in the $\left(\xi ,\eta \right)$ system are given by:

(31) $$\omega =-{\left[\left(Q^{2}+D^{2}\right)\frac{\partial^{2}\psi }{\partial \eta^{2}}\right]}_{\eta =0,1}$$

The wall boundary conditions for θ and φ in the $\left(\xi ,\eta \right)$ system are given by:

(32) $$\begin{array}{cc} \theta =\varphi ={\left.1\right|}_{\eta =0}, & \theta =\varphi ={\left.0\right|}_{\eta =1} \end{array}$$

At the outlet, the boundary conditions for all the variables are used as the fully developed flow. The flow is defined as sinusoidal for the pulsating flow:

(33) $$\begin{array}{cc} u\left(y,t\right)=u\left(y\right)\left[1+\sin\left(2\pi t\right)\right], & v=0 \end{array}$$

Furthermore, no-slip conditions are considered on the walls.

### 2.5. Transformation of Coordinates

The constricted part of the channel wall is mapped to a straight line. Consider the following coordinate transformation

(34) $$\begin{array}{cc} \xi =x, & \eta =\frac{y-y_{1}\left(x\right)}{y_{2}\left(x\right)-y_{1}\left(x\right)} \end{array}$$

After this transformation, the lower and the upper walls of the channel are represented by η=0 and η=1, respectively, and Equations (20) and (21), (24) and (25) in the new coordinate system $\left(\xi ,\eta \right)$ are given as

(35) $$St\frac{\partial \omega }{\partial t}+u\left(\frac{\partial \omega }{\partial \xi }-Q\frac{\partial \omega }{\partial \eta }\right)+vD\frac{\partial \omega }{\partial \eta }\;=\frac{1}{\emptyset_{3}}\frac{1}{Re}\left(1+\frac{1}{\beta }\right)\left[\frac{\partial^{2}\omega }{\partial \xi^{2}}-\left(P-2QR\right)\frac{\partial \omega }{\partial \eta }-2Q\frac{\partial^{2}\omega }{\partial \xi \partial \eta }+\left(Q^{2}+D^{2}\right)\frac{\partial^{2}\omega }{\partial \eta^{2}}\right]+\frac{1}{\emptyset_{1}}\frac{M^{2}}{Re}D^{2}\frac{\partial^{2}\psi }{\partial \eta^{2}}+ReD_{a}^{2}\omega$$

(36) $$\frac{\partial^{2}\psi }{\partial \xi^{2}}-\left(P-2QR\right)\frac{\partial \psi }{\partial \eta }-2Q\frac{\partial^{2}\psi }{\partial \xi \partial \eta }+\left(Q^{2}+D^{2}\right)\frac{\partial^{2}\psi }{\partial \eta^{2}}=-\omega$$

(37) $$St\frac{\partial \theta }{\partial t}+u\left(\frac{\partial \theta }{\partial \xi }-Q\frac{\partial \theta }{\partial \eta }\right)+vD\frac{\partial \theta }{\partial \eta }=\frac{1}{Peh}\frac{\emptyset_{5}}{\emptyset_{4}}\left[\frac{\partial^{2}\theta }{\partial \xi^{2}}-\left(P-2QR\right)\frac{\partial \theta }{\partial \eta }-2Q\frac{\partial^{2}\theta }{\partial \xi \partial \eta }+\left(Q^{2}+\left(1+\frac{Rd}{\emptyset_{5}}\right)D^{2}\right)\frac{\partial^{2}\theta }{\partial \eta^{2}}\right]$$

(38) $$St\frac{\partial \varphi }{\partial t}+u\left(\frac{\partial \varphi }{\partial \xi }-Q\frac{\partial \varphi }{\partial \eta }\right)+vD\frac{\partial \varphi }{\partial \eta }=\frac{1}{Pem}\left[\frac{\partial^{2}\varphi }{\partial \xi^{2}}-\left(P-2QR\right)\frac{\partial \varphi }{\partial \eta }-2Q\frac{\partial^{2}\varphi }{\partial \xi \partial \eta }+\left(Q^{2}+D^{2}\right)\frac{\partial^{2}\varphi }{\partial \eta^{2}}\right]\;+\frac{Sr}{Re}\left[\frac{\partial^{2}\theta }{\partial \xi^{2}}-\left(P-2QR\right)\frac{\partial \theta }{\partial \eta }-2Q\frac{\partial^{2}\theta }{\partial \xi \partial \eta }+\left(Q^{2}+D^{2}\right)\frac{\partial^{2}\theta }{\partial \eta^{2}}\right]$$

where

$$\begin{array}{cc} P=P\left(\xi ,\eta \right)=\frac{\eta y_{2}^{\prime \prime }\left(\xi \right)+\left(1-\eta \right)y_{1}^{\prime \prime }\left(\xi \right)}{y_{2}\left(\xi \right)-y_{1}\left(\xi \right)}, & Q=Q\left(\xi ,\eta \right)=\frac{\eta y_{2}^{\prime }\left(\xi \right)+\left(1-\eta \right)y_{1}^{\prime }\left(\xi \right)}{y_{2}\left(\xi \right)-y_{1}\left(\xi \right)},\\ R=R\left(\xi \right)=\frac{y_{2}^{\prime }\left(\xi \right)-y_{1}^{\prime }\left(\xi \right)}{y_{2}\left(\xi \right)-y_{1}\left(\xi \right)}, & D=D\left(\xi \right)=\frac{1}{y_{2}\left(\xi \right)-y_{1}\left(\xi \right)} \end{array}$$

where the velocity components u and v in terms of $\left(\xi ,\eta \right)$ take the forms

$$\begin{array}{cc} u=D\left(\xi \right)\frac{\partial \psi }{\partial \eta }, & v=Q\left(\xi ,\eta \right)\frac{\partial \psi }{\partial \eta }-\frac{\partial \psi }{\partial \xi } \end{array}$$

The non-dimensional physical quantities, Nusselt number $\left(Nu\right)$, Sherwood $\left(Sh\right)$, and skin friction coefficient $\left(C_{f}\right)$, are defined as

$$\begin{array}{ccc} Nu=-\left(\frac{L}{k_{f}\left(T_{1}-T_{2}\right)}\right)\left(k_{hnf}+\frac{16\sigma T_{\infty }^{3}}{3k^{*}}\right){\left.\frac{\partial \tilde{T}}{\partial \tilde{y}}\right|}_{\tilde{y}=0}, & C_{f}=\frac{\tau_{w}}{\rho_{f}u_{w}^{2}}, & Sh=\frac{-hc_{w}}{\left(C_{1}-C_{2}\right)} \end{array}$$

where $\tau_{w}$, and $c_{w}$ are defined as

$$\begin{array}{cc} \tau_{w}={\left[\left(\mu_{hnf}\left(1+\frac{1}{\beta }\right)\right)\frac{\partial \tilde{u}}{\partial \tilde{y}}\right]}_{\tilde{y}=0}, & c_{w}={\left[\frac{\partial \tilde{C}}{\partial \tilde{y}}\right]}_{\tilde{y}=0} \end{array}$$

After using dimensionless variables from Equation (10) and the coordinate transformation from Equation (34), we get

$$C_{f}=\frac{1}{u_{w}^{2}}{\left[-\frac{1}{Re\emptyset_{3}}\left(1+\frac{1}{\beta }\right)D\frac{\partial u}{\partial \eta }\right]}_{\eta =0}$$

$$Nu=-\left[\frac{k_{hnf}}{k_{f}}\left(1+Rd\right)\theta^{\prime }\left(0\right)\right]=-\left[\emptyset_{5}\left(1+Rd\right)\theta^{\prime }\left(0\right)\right]$$

$$Sh={\left[-D\frac{\partial \varphi }{\partial \eta }\right]}_{\eta =0}$$

where $Rd=\frac{16\sigma T_{\infty }^{3}}{3k^{*}k_{hnf}}$ and $\emptyset_{5}=\frac{k_{hnf}}{k_{f}}$.

## 3. Numerical Scheme and Validation

The system (35)–(38) subject to the relevant boundary conditions is solved numerically as in [6,7,8,9,10,41,42]. We consider the computation domain as −10≤ξ≤10 and 0≤η≤1. For a stable solution, the domain is discretized using the step size of Δξ=0.05 and Δη=0.02 in ξ and η directions, thus forming a Cartesian grid of 400×50 elements. The height of constriction at both walls is considered as 0.35, and the Reynolds number is taken as 800 unless specified otherwise. Moreover, time step size Δt=0.00005 is considered for the flow with pulsation. The solution at time level l is specified, but the solution at each time level l+1, for l=0,1,2,⋯, is computed. Using the well-known TDMA (Tri-Diagonal Matrix Algorithm) and central differences for the discretization of the space derivatives, Equation $\left(36\right)$ is solved for $\psi =\psi \left(\xi ,\eta \right)$. Whereas, using the ADI (Alternating Direction Implicit) scheme, Equations (35) and (37), (38) are solved for $\omega =\omega \left(\xi ,\eta \right)$, $\theta =\theta \left(\xi ,\eta \right)$, and $\varphi =\varphi \left(\xi ,\eta \right)$, respectively. The forward/backward difference is used to discretize the time derivative, and the central differences are used to discretize the space derivatives. The computations for the present study are performed in a sequential fashion. The results can be found by parallel computing for time-efficient solutions; see Ali and Syed [43].

The pulsatile flows were simulated over a long enough period of time to avoid transition effects on the solution; i.e., a steady periodicity in the flow is established. For this purpose, we choose the sixth time interval that has been used to compute these data. The results are displayed at the four time instants of the pulse cycle; i.e., t=0, 0.25, 0.5, 0.75. At t=0, the pulse cycle is started. For 0<t<0.25, the flow accelerates, and its peak is at t=0.25. For 0.25<t<0.5, the flow rate decreases and reaches its minimum at t=0.5. At t=0.75, the instantaneous flow rate becomes zero [6,10,42]. Moreover, to analyze the profiles at the prominent x locations, the results are displayed at selected time instants and axial locations. We perform simulations for a long enough time; however, in most cases, the results are displayed at selected time instants and axial locations, especially x=0 (throat of the constriction) and x=2 (in the lee of the constriction) where the fluid enters the low-pressure zone from the high-pressure zone.

**Figure 2:**
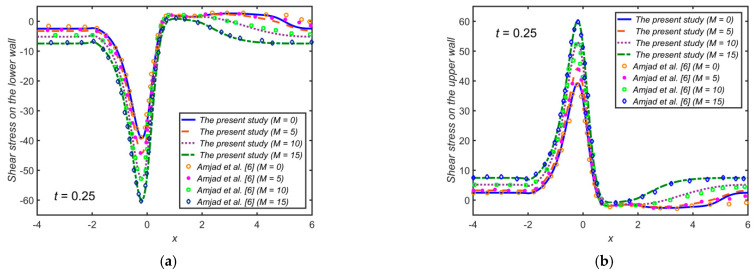
The WSS distribution for distinct values of the Hartmann number M (**a**) on the lower wall; (**b**) on the upper wall.

For validation, the present results for the pulsatile flow of the base fluid (i.e., with $\phi_{1}=0,\;\phi_{2}=0$) are compared with those obtained by Amjad et al. [6] in the case of Casson fluid flow. Figure 2 shows a good agreement of the present results, specifically the WSS, with [6] for M=0, 5, 10, 15 at t=0.25.

## 4. Results and Discussion

This section deals with the physical influence of constraints on the NF with Cu NPs and HNF with Cu-SWCNT NPs when M=5, St=0.02, β=0.5, $D_{a}=0.002$, Re=800, Rd=0.2, Peh=490, Pem=420, Sr=0.8, and $\phi_{1}=\phi_{2}=0.03$. The foregoing values are kept constant in the entire article except when we explicitly mention otherwise. The initial guesses for dependent variables are selected using the hit and trial method until the correct asymptotic behavior of the solution for Cu-based NF is achieved.

### 4.1. The Magnetic Parameter Effect

**Figure 3:**
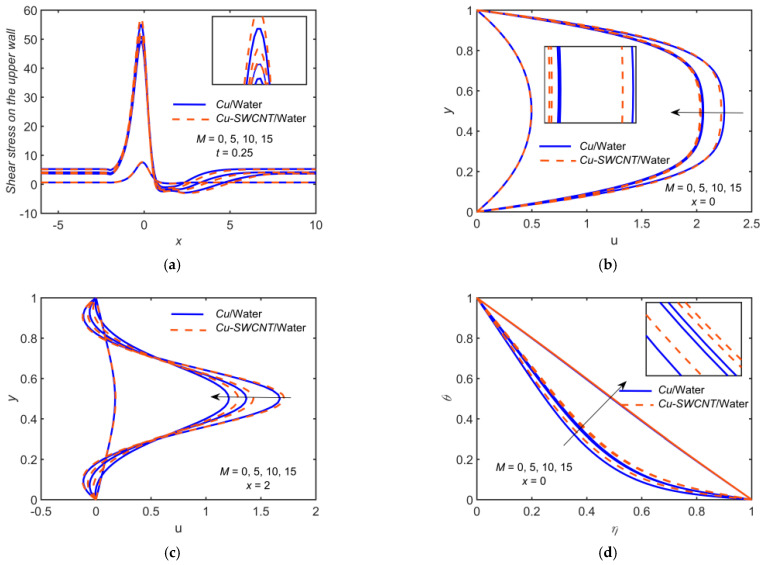

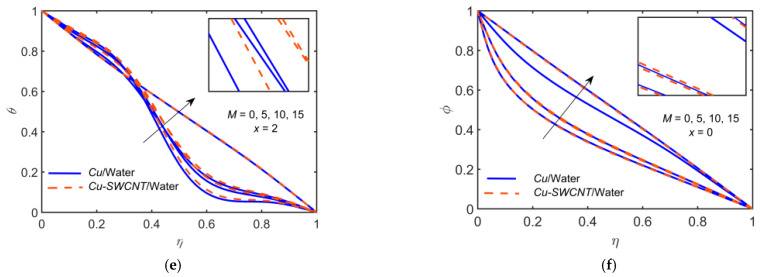
(**a**) The WSS distribution, (**b**) u profile at x=0, (**c**) u profile at x=2, (**d**) *θ* profile at x=0, (**e**) *θ* profile at x=2, and (**f**) *φ* profile at x=0 for distinct values of M.

Figure 3 depicts the impact of Cu-based NF and Cu-SWCNT-based HNF for Hartman number M=0, 5, 10, 15 on (a) the WSS distribution on the upper wall, (b) u profile at x=0, (c) u profile at x=2, (d) the temperature $\left(\theta \right)$ profile at x=0, (e) the temperature $\left(\theta \right)$ profile at x=2, and (f) the concentration $\left(\varphi \right)$ profile at x=0. Figure 3a shows that as M estimations intensify, the WSS on the upper wall escalates, and it is maximum at t=0.25. The WSS for HNF is higher as compared to NF. Figure 3b,c show that the velocity field’s boundary layer thickness escalates as M grows. This is due to the fact that the Lorentz force measures the presence of M, so a delaying force is conceived in the velocity field. As Hartmann number estimations grow, the restricting force increases, and the velocity field reduces. The HNF velocity profile is slightly lower than that of the NF at x=0. The profiles are not parabolic, as some backflow in the vicinity of the walls is observed at x=2, so in this case, the velocity profile for HNF is higher as compared to NF. The backflow reduces with an increase in M. Figure 3d–f show the opposite effects of M on the temperature and concentration fields. The Hartmann number has an inverse relation toward the density of HNF. So, it is found that as the estimations of M grow, the fluid density shrinks and the temperature increases. Hence, escalation in M shrinks the fluid density, which causes temperature rises. The HNF temperature profile is slightly higher than that of the NF.

### 4.2. The Pulsation Parameter Effect

**Figure 4:**
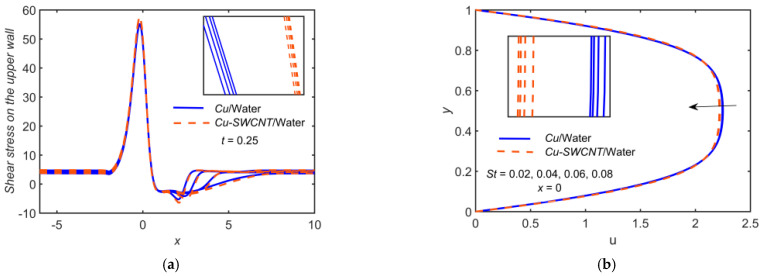

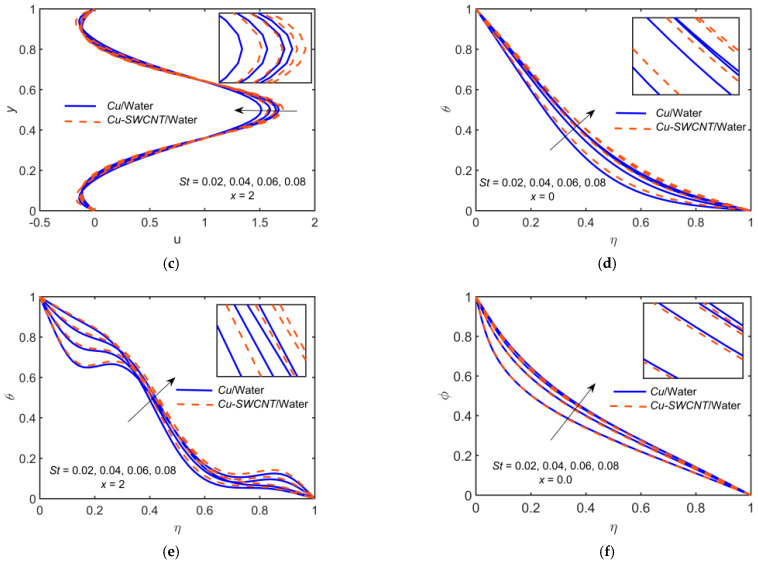
(**a**) The WSS distribution, (**b**) u profile at x=0, (**c**) u profile at x=2, (**d**) θ profile at x=0, (**e**) θ profile at x=2, and (**f**) φ profile at x=0 for distinct values of St.

Figure 4 depicts the impact of Cu-based NF and Cu-SWCNT-based HNF for Strouhal number (pulsation parameter) St=0.02, 0.04, 0.06, 0.08 on (a) the WSS distribution on the upper wall, (b) u profile at x=0, (c) u profile at x=2, (d) the temperature $\left(\theta \right)$ profile at x=0, (e) the temperature $\left(\theta \right)$ profile at x=2, and (f) the concentration $\left(\varphi \right)$ profile at x=0. Figure 4a shows that as the St estimations intensify, the WSS on the upper wall escalates, and it is maximum at t=0.25. The WSS for HNF is higher as compared to NF. The velocity field’s boundary layer thickness escalates as St grows, and the velocity field reduces, as shown in Figure 4b,c. The u profile coincides for all the values of St, and the HNF velocity profile is slightly lower than that of the NF when x=0. The profiles are not parabolic, as some backflow in the vicinity of the walls is observed at x=2, so in this case, the velocity profile for HNF is higher as compared to NF. Figure 4d–f show the opposite effects of St on the temperature and concentration fields. It is found that as the estimations of St grow, the temperature at the surface and the thermal boundary layer thickness reduces. The HNF θ profile is slightly higher than that of the NF for temperature profiles, but it is opposite in the case of the φ profile.

### 4.3. The Casson Fluid Parameter Effect

**Figure 5:**
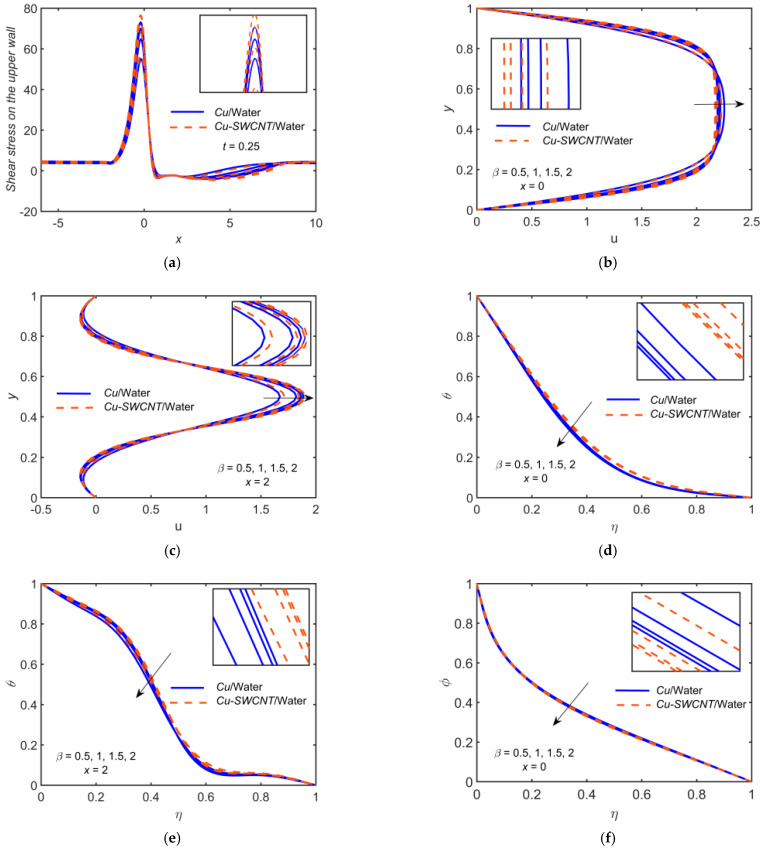
(**a**) The WSS distribution, (**b**) u profile at x=0, (**c**) u profile at x=2, (**d**) θ profile at x=0, (**e**) θ profile at x=2, and (**f**) φ profile at x=0 for distinct values of β.

Figure 5 depicts the impact of Cu-based NF and Cu-SWCNT-based HNF for Casson fluid parameter β=0.5, 1, 1.5, 2 on (a) the WSS distribution on the upper wall, (b) the u profile at x=0, (c) the u profile at x=2, (d) the temperature $\left(\theta \right)$ profile at x=0, (e) the temperature $\left(\theta \right)$ profile at x=2, and (f) the concentration $\left(\varphi \right)$ profile at x=0. Figure 5a shows that as β estimations intensify, the WSS on the upper wall escalates, and it is maximum at t=0.25. The WSS for HNF is higher as compared to NF. The velocity field’s boundary layer thickness reduces as β grows, and the velocity field escalates, as shown in Figure 5b,c. The reason for this behavior is that as the value of β grows, the elasticity of HNF rises, causing the HNF to become more viscous. Physically, the boundary layer thickness in such a case reduces as β grows. The HNF velocity profile is slightly lower than that of the NF when x=0, but an opposite behavior was noticed when x=2. Figure 5d–f show that the θ and φ profiles have a declining behavior toward β. The HNF temperature profile is slightly higher than that of the NF for temperature profiles but opposite in the case of concentration profile.

### 4.4. The Porosity Parameter Effect

**Figure 6:**
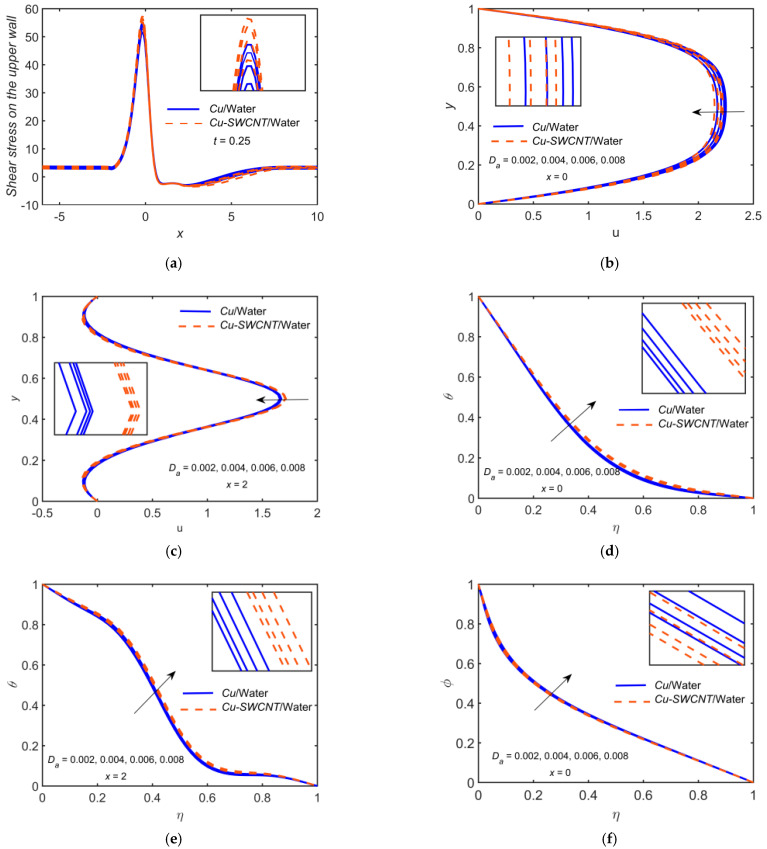
(**a**) The WSS distribution, (**b**) u profile at x=0, (**c**) u profile at x=2, (**d**) θ profile at x=0, (**e**) θ profile at x=2, and (**f**) φ profile at x=0 for distinct values of $D_{a}$.

Figure 6 depicts the impact of Cu-based NF and Cu-SWCNT-based HNF for porosity parameter $D_{a}=0.002,\;0.004,\;0.006,\;0.008$ on (a) the WSS distribution on the upper wall, (b) u profile at x=0, (c) u profile at x=2, (d) the temperature $\left(\theta \right)$ profile at x=0, (e) the temperature $\left(\theta \right)$ profile at x=2, and (f) the concentration $\left(\varphi \right)$ profile at x=0. Figure 6a shows that as $D_{a}$ estimations intensify, the WSS on the upper wall reduces. The WSS for HNF is higher as compared to NF. Figure 6b,c show that the velocity field’s boundary layer thickness escalates as $D_{a}$ grows and the velocity field reduces. The HNF velocity profile is slightly lower than that of the NF when x=0, but opposite behavior is noticed when x=2. Figure 6d–f show that the θ and φ profiles escalate as $D_{a}$ grows. The HNF temperature profile is slightly higher than that of the NF for temperature profiles but opposite in the case of concentration profile.

### 4.5. The Reynolds Number Effect

**Figure 7:**
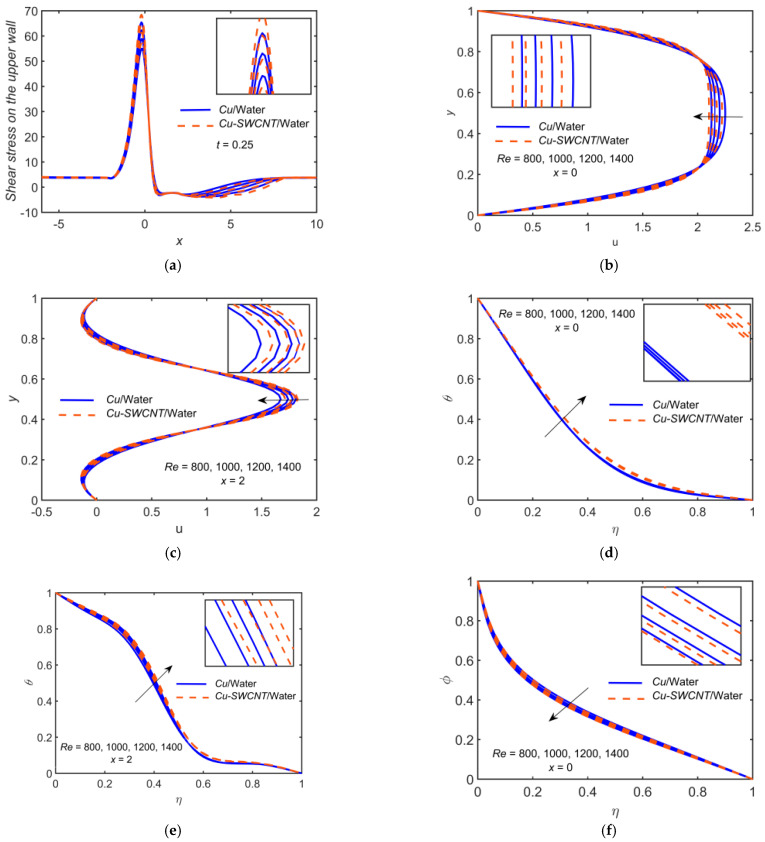
(**a**) The WSS distribution, (**b**) u profile at x=0, (**c**) u profile at x=2, (**d**) θ profile at x=0, (**e**) θ profile at x=2, and (**f**) φ profile at x=0 for distinct values of Re.

Figure 7 depicts the impact of Cu-based NF and Cu-SWCNT-based HNF for Reynolds number Re=800, 1000, 1200, 1400 on (a) the WSS distribution on the upper wall, (b) the u profile at x=0, (c) the u profile at x=2, (d) the temperature $\left(\theta \right)$ profile at x=0, (e) the temperature $\left(\theta \right)$ profile at x=2, and (f) the concentration $\left(\varphi \right)$ profile at x=0. Figure 7a shows that as Re estimations intensify, the WSS on the upper wall escalates. As expected, the magnitudes of the peak values of the profiles are lower for the lower Re. The WSS for HNF is higher compared to NF. Figure 7b,c show that the velocity field’s boundary layer thickness escalates as Re grows and the velocity field reduces. The reason for this condition is the formation of a thinner boundary layer with the increase of Re. The HNF velocity profile is slightly lower than that of the NF when x=0, but the opposite behavior is noticed when x=2. Figure 7d–f shows that the θ profile escalates but the φ profile reduces as Re grows. The HNF temperature profile is slightly higher than that of the NF for temperature profiles but opposite in the case of concentration profile. This may be due to the shear thinning nature of HNF, where effective viscosity becomes almost constant at a high shear rate. It may be noted that at higher Re, any shear thinning fluid behaves similar to Newtonian fluid. In fact, escalating values of Re reduce the internal energy of NPs, which lowers the concentration profile.

### 4.6. The Varying Time Effect

**Figure 8:**
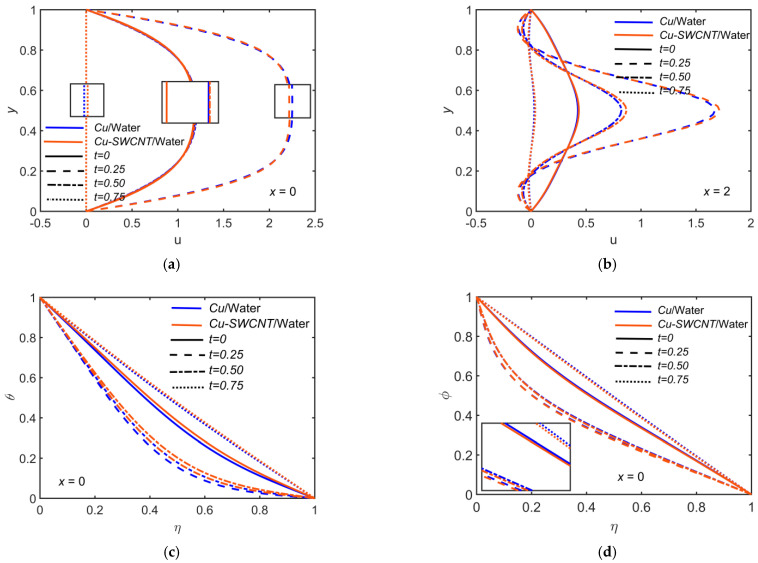
(**a**) u profile at x=0, (**b**) u profile at x=2, (**c**) θ profile at x=0, and (**d**) φ profile at x=0 for distinct values of t.

Figure 8 depicts the impact of Cu-based NF and Cu-SWCNT-based HNF for time t=0, 0.25, 0.50, 0.75 on (a) the u profile at x=0, (b) the u profile at x=2, (c) the θ profile at x=0, and (d) the φ profile at x=0. Figure 8a,b show that for the HNF, the particle concentration in the fluid is even higher than that of the NF, which causes an enhancement of the density and dynamic viscosity. The flow velocity experiences reductions accordingly. At x=0, the maximum value of u grows, and the curves appear to be parabolic for 0≤t≤0.5. At t=0.75, u is dropped substantially. At x=2, which is the vicinity of the limiting point of the constriction downstream, backflow occurs near the walls. The HNF velocity profile is slightly lower than that of the NF when x=0, but opposite behavior is noticed when x=2. Figure 8c shows that the temperature profile escalates for HNF compared to NF as the values of t upsurge. A rapid growth in temperature is due to the hybrid nature of NF, since it escalates the thermal conductivity. Figure 8d shows the opposite behavior in the case of concentration profile.

### 4.7. The Radiation Parameter Effect

**Figure 9:**
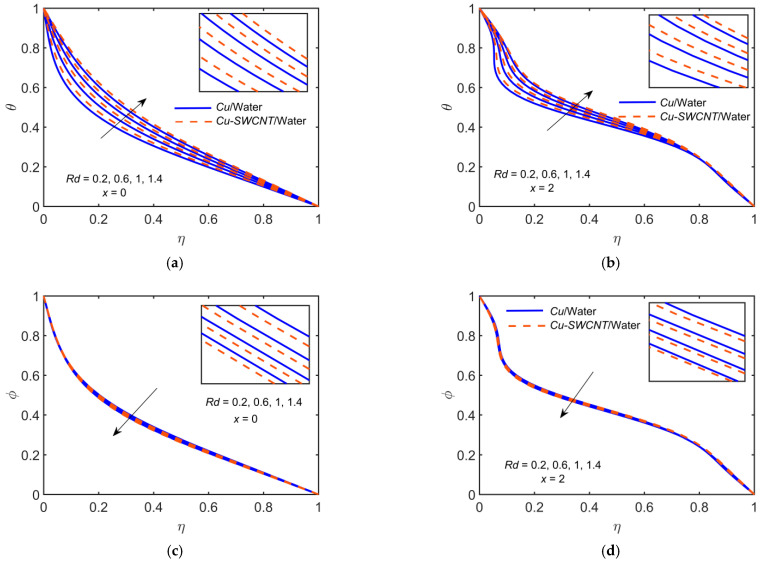
(**a**) θ profile at x=0, (**b**) θ profile at x=2, (**c**) φ profile at x=0, and (**d**) φ profile at x=2 for distinct values of Rd.

Figure 9 depicts the impact of Cu-based NF and Cu-SWCNT-based HNF for Radiation parameter Rd=0.2, 0.6, 1, 1.4 on (a) the temperature $\left(\theta \right)$ profile at x=0, (b) the temperature $\left(\theta \right)$ profile at x=2, (c) the concentration $\left(\varphi \right)$ profile at x=0, and (d) the concentration $\left(\varphi \right)$ profile at x=2. Figure 9a,b show that as Rd estimations intensify, the HNF thermal distribution is improved. Basically, the values of Rd provide extra heat to NF, which results in the rise of the temperature profile. Theoretically, thermal radiation causes an escalation in the amount of heat on the surface. This means that the fluid absorbs more heat from the radiation. Heat is dissipated away from the surface, raising the temperature profile of HNFs. In general, as Rd estimations intensify, it escalates the electromagnetic radiation and, as a result, the fluid’s hotness. Figure 9c,d show that the concentration profiles reduce as Rd escalates. The HNF temperature profile is slightly higher than that of the NF for temperature profiles but opposite in the case of concentration profile.

### 4.8. The Solid Volume Fraction Effect

**Figure 10:**
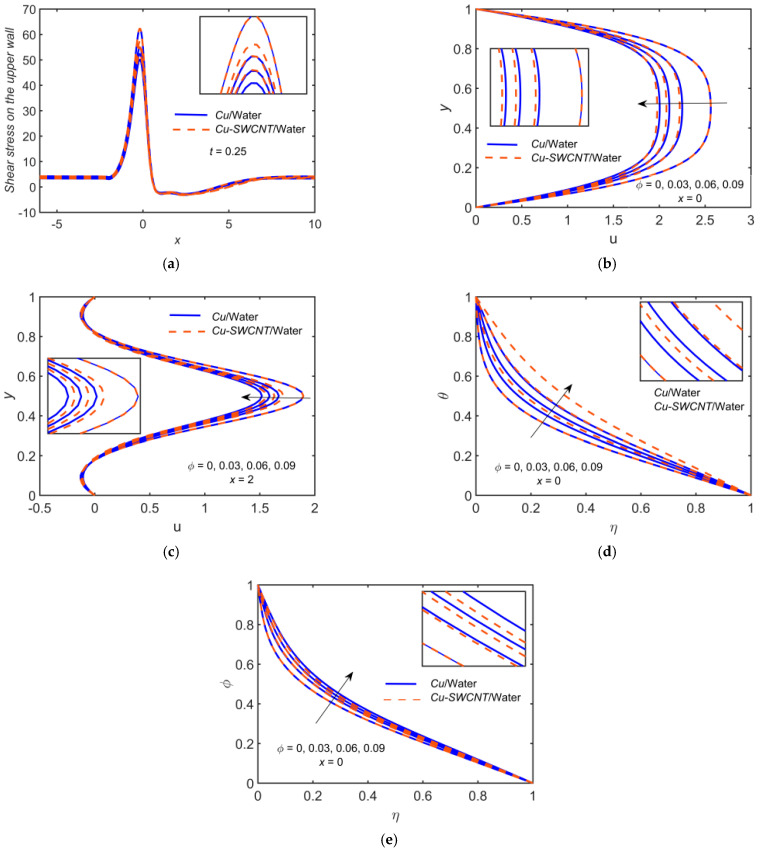
(**a**) The WSS distribution, (**b**) u profile at x=0, (**c**) u profile at x=2, (**d**) θ profile at x=0, and (**e**) φ profile at x=0 for distinct values of ϕ.

Figure 10 depicts the impact of Cu-based NF and Cu-SWCNT-based HNF for the solid volume fraction ϕ=0, 0.03, 0.06, 0.09 on (a) the WSS distribution on the upper wall, (b) the u profile at x=0, (c) the u profile at x=2, (d) the temperature $\left(\theta \right)$ profile at x=0, and (e) the concentration $\left(\varphi \right)$ profile at x=0. Figure 7a shows that as ϕ estimations intensify, the WSS on the upper wall reduces. There is no significant impact observed of varying ϕ by comparing HNF and NF. Figure 7b,c show that the velocity field reduces. Physically, the momentum boundary layer thickness tends to decrease with upsurging ϕ. Thus, the velocity field’s boundary layer thickness escalates as ϕ grows. The HNF velocity profile is slightly lower than that of the NF when x=0, but the opposite behavior is noticed when x=2 where backflow is observed. Figure 7d–f show that the temperature and concentration profiles escalate as ϕ grows. When looking at the graphs, it is clear that the convection heat transfer coefficient escalates as the volume fraction of NPs grows in all HNFs. This is due to the fact that the fraction of NPs affects the thermophysical properties of the base fluid and improves the convective heat transfer performance. In addition, the thermal conductivity and thermal boundary layer thickness reduce with escalating ϕ values. The HNF temperature profile is slightly higher than that of the NF for temperature profiles but opposite in the case of concentration profile.

### 4.9. Some Other Physical Parameters Effect

**Figure 11:**
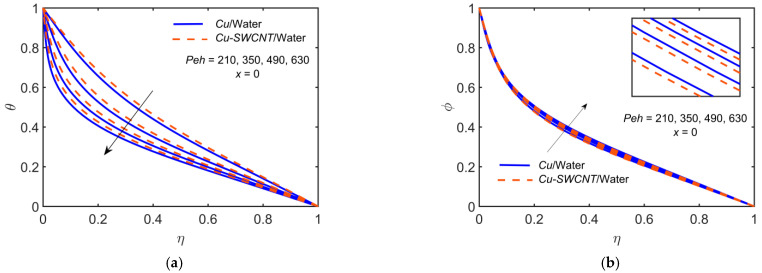
(**a**) The θ profile and (**b**) φ profile at x=0 for distinct values of Peh.

Figure 11 depicts the impact of Cu-based NF and Cu-SWCNT-based HNF for the heat diffusion parameter Peh=210, 350, 490, 630 on (a) the temperature $\left(\theta \right)$ profile at x=0, and (b) the concentration $\left(\varphi \right)$ profile at x=0. This figure shows that as Peh estimations intensify, the temperature profile reduces, and the concentration profile escalates. The Peclet number shows a high impact on the microorganism’s density in a blood NF. The HNF temperature profile is slightly higher than that of the NF for temperature profiles but opposite in the case of concentration profile.

**Figure 12:**
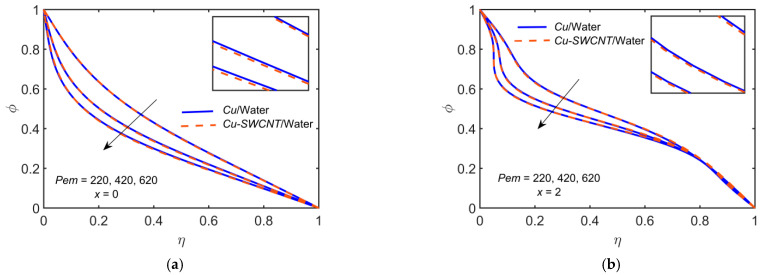
The φ profile at (**a**) x=0 and (**b**) x=2 for distinct values of Pem.

Figure 12 depicts the impact of Cu-based NF and Cu-SWCNT-based HNF for the mass diffusion parameter Pem=220, 420, 620 on (a) the concentration $\left(\varphi \right)$ profile at x=0 and (b) the concentration $\left(\varphi \right)$ profile at x=2. The figure shows that as Pem estimations intensify, the concentration profile reduces. The HNF concentration profile is slightly lower than that of the NF.

**Figure 13:**
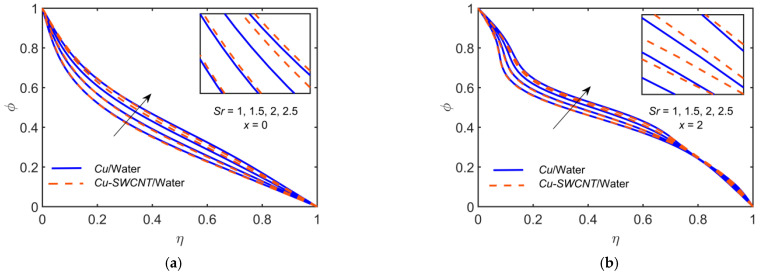
The φ profile at (**a**) x=0 and (**b**) x=2 for distinct values of Sr.

Figure 13 depicts the impact of Cu-based NF and Cu-SWCNT-based HNF for the Soret number Sr=1, 1.5, 2, 2.5 on (a) the concentration $\left(\varphi \right)$ profile at x=0, and (b) the concentration $\left(\varphi \right)$ profile at x=2. The figure shows that as Sr estimations intensify, the concentration profile escalates. The HNF concentration profile is slightly higher than that of the NF.

### 4.10. The Influence of Physical Parameters on the Nusselt Number, Sherwood Number, and Skin Friction Coefficient Profiles

**Figure 14:**
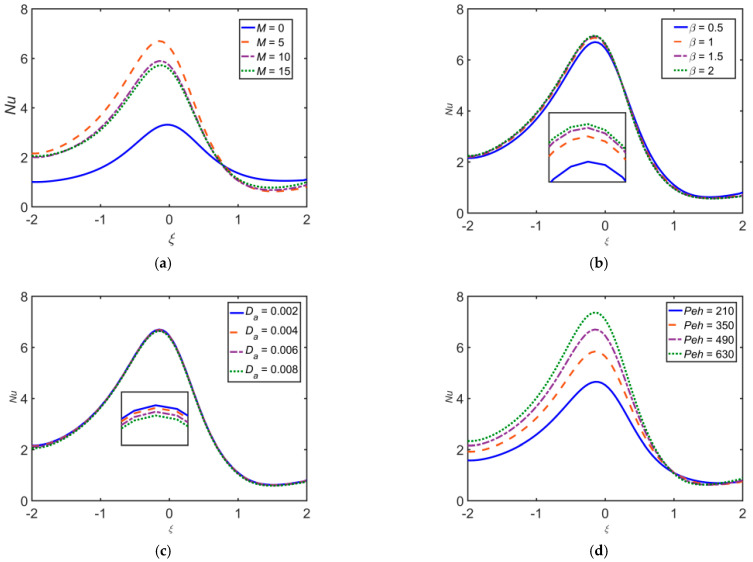

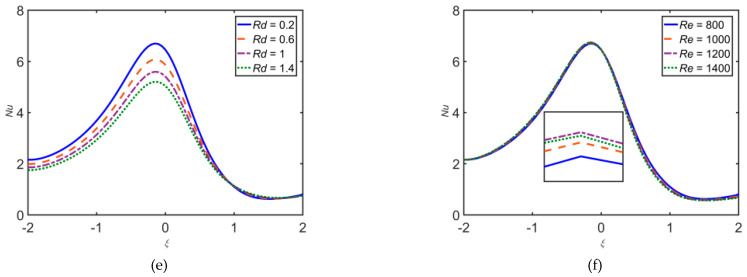
Impact of (**a**) M, (**b**) β, (**c**) $D_{a}$, (**d**) Peh, (**e**) Rd, and (**f**) Re on Nu.

Figure 14 depicts the effect of various flow governing parameters on Nusselt number Nu. The Nu escalates as β, Re, and Peh estimations intensify. Nu reduces upon escalating M, $D_{a}$, and Rd.

**Figure 15:**
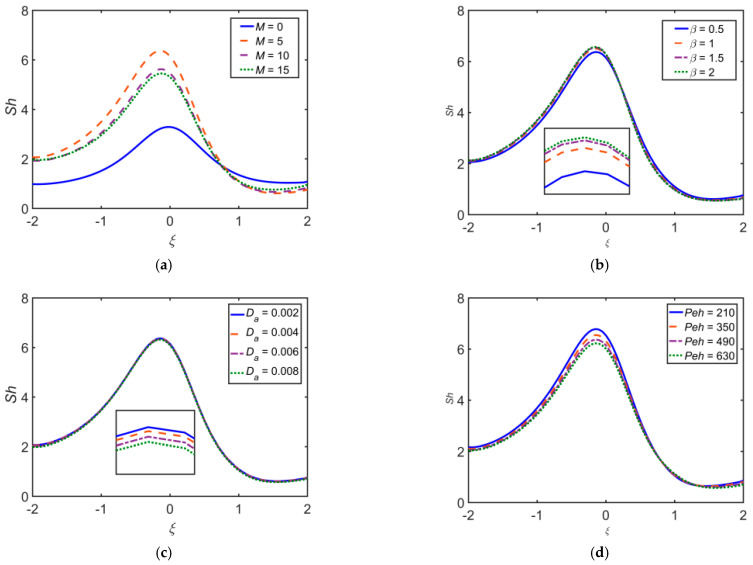

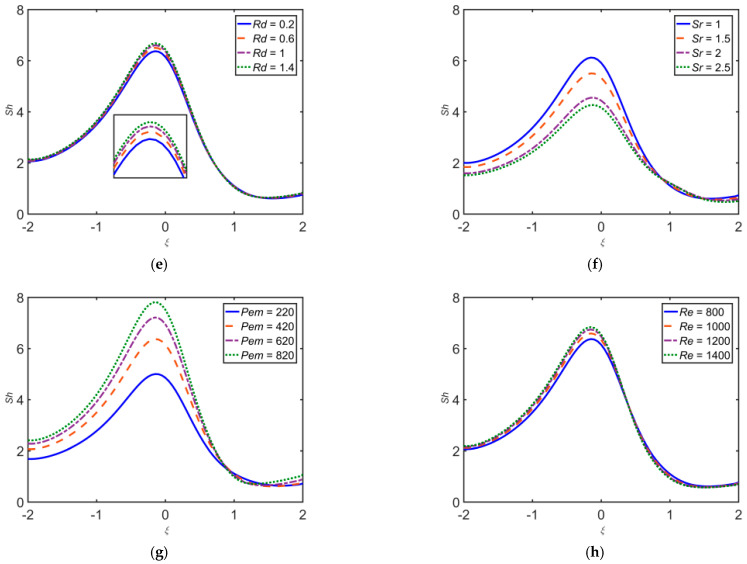
Impact of (**a**) M, (**b**) β, (**c**) $D_{a}$, (**d**) Peh, (**e**) Rd, (**f**) Sr, (**g**) Pem, and (**h**) Re on Sh.

Figure 15 depicts the influence of different flow governing parameters on the Sherwood number Sh. It is observed that Sh escalates as β, Rd, Re, and Pem estimations intensify, whereas Sh reduces upon escalating $D_{a}$, M, Peh, and Sr.

**Figure 16:**
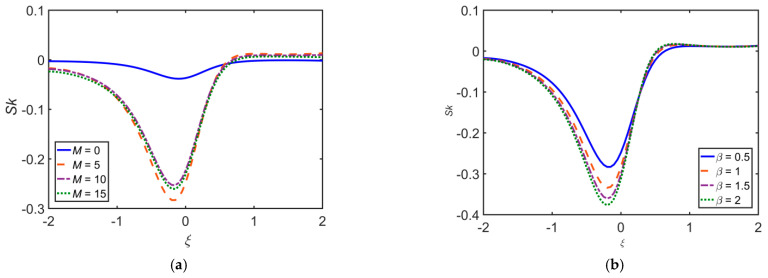

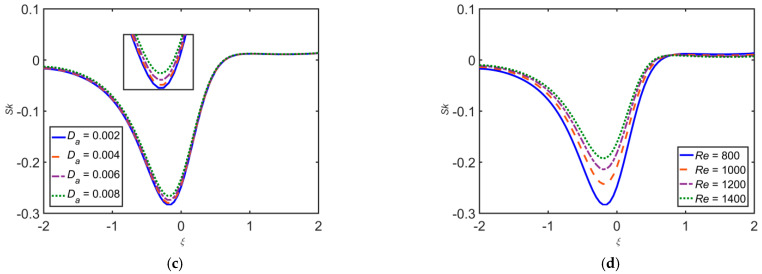
Impact of (**a**) M, (**b**) β, (**c**) $D_{a}$, and (**d**) Re on Sk.

Figure 16 depicts the effect of various flow governing parameters on skin friction coefficient Sk. The Sk has a direct relation with $D_{a}$ and Re, whereas it has an inverse relation with M and β.

The Nusselt number grows as the flow becomes more turbulent due to the rising number of collisions among the fluid particles. Since more viscous fluids have a lower Reynolds number, less heat transfer occurs, lowering the Nusselt number. The Nusselt number grows as β estimations intensify.

**Figure 17:**
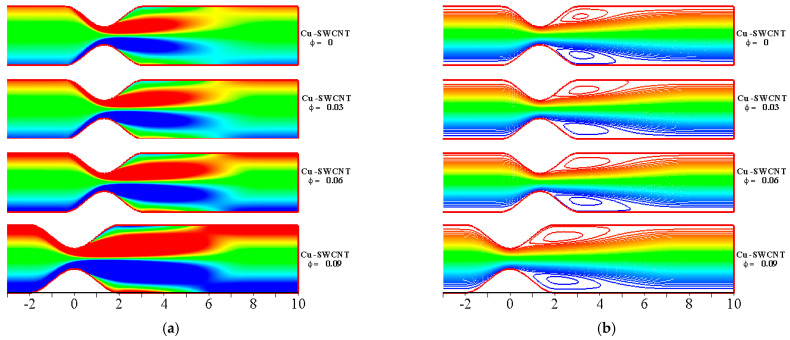

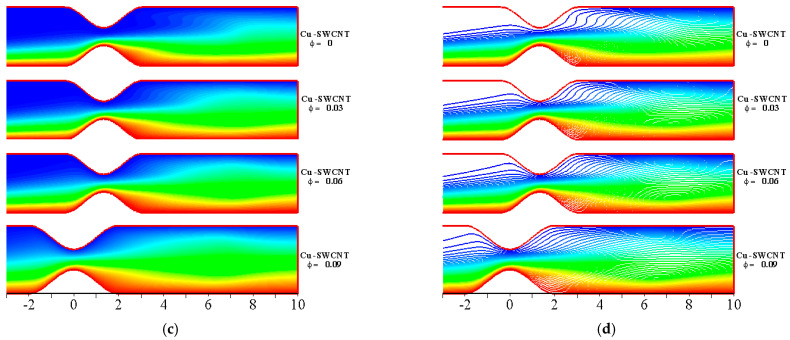
The impact of HNF on (**a**) vorticity, (**b**) streamlines, (**c**) temperature distributions, and (**d**) concentration profile for distinct values of ϕ with Re=800, M=5, St=0.02, and Rd=0.2, at the maximum flow rate (t=0.25).

The streamlines, vorticity, temperature distribution, and concentration plots with M=5, St=0.02, Rd=0.2, and Re=800 are shown in Figure 17 for Cu-SWCNT-based HNF. The formation of vertical eddies in the vicinity of the walls can be observed. The eddies increase for the HNF and slowly occupy a major part of the channel downstream of the constriction. The inclusion of multiple types of NPs adds more energy. This results in higher increments in the temperature as well as the thickness of the thermal boundary layer. A rapid rise in the temperature is due to the HNF, since it increases the thermal conductivity. However, the significant impact can be seen in the case of concentration profile.

## 5. Concluding Remarks

In this research, heat and mass transfer analysis of the pulsatile flow of a Casson hybrid nanofluid through a constricted channel in the presence of viscous incompressible MHD pulsatile fluid flow over a rectangular channel is numerically investigated. The impact of flow governing parameters such as the magnetic parameter, Casson parameter, Reynolds number, Strouhal number, porosity parameter, radiation parameter, Peclet number for the diffusion of heat and mass, and Soret number on the WSS, u, θ, and φ profiles for the sake of comparison between the behavior of Cu-based NF and Cu-SWCNT-based HNF are analyzed in the form of graphs. The physical quantities and heat and mass transfer coefficients are also calculated for the present model, and the impact of flow controlling parameters on these quantities is graphically analyzed. The key findings can be summarized as follows.

(1) The WSS escalates with the rising value of M, St, β, and Re, whereas it reduces with rising values of Da and ϕ. The WSS for HNF is observed to be the highest, which is followed by NF.
(2) The velocity field’s boundary layer thickness escalates as M, St, $D_{a}$, Re, and ϕ grow, and the velocity field reduces but shows opposite behavior for β. As Hartmann number estimations grow, the restricting force increases, and the velocity field reduces. The HNF velocity profile is slightly lower than that of the NF when x=0, but opposite behavior is noticed when x=2 in the case of ϕ.
(3) The research shows that a magnetic field applied to the blood reduces the velocity of both blood and magnetic particles.
(4) Temperature profiles escalate as M, St, $D_{a}$, Re, Rd, and ϕ grow but reduce as β, and Peh estimations intensify.
(5) A rapid rise in temperature is observed due to the HNF, since it increases the thermal conductivity when comparing the NF and HNF. Hence, it is interpreted that the HNF hits higher temperatures compared to the NF.
(6) The concentration profile escalates as M, St, $D_{a}$, Peh, Sr, and ϕ grow but reduces as β, Re, Rd, Pem and Peh estimations intensify.
(7) The heat transfer rate, Sherwood number, and skin friction coefficient escalate as Re estimations intensify and reduce by escalating the porosity parameter. The Nusselt number grows as β estimations intensify.

This research might provide a good picture of the heat and mass transfer activity of blood supply in a circulatory system for treatment such as multiple hyperthermia therapies.

## Figures and Tables

**Table 1:** Thermophysical properties of the base fluid water and the two kinds of NPs [38,39].

Physical Property	Base Fluid	Nanoparticles	
	$H_{2}O$	**Cu**	**SWCNT**
$C_{p}$ (J/kgK)	4179	385	235
ρ (kg/m^3^)	997.1	8933	10500
k (W/mK)	0.613	401	429

## Data Availability

Not applicable.

## Nomenclature

$y_{1}$	constricted channel’s lower wall
$y_{2}$	constricted channel’s upper wall
$h_{1}$	upper wall constriction height
$h_{2}$	lower wall constriction height
π	product of deformation rate’s component
$\pi_{c}$	critical value of π
$p_{y}$	yield stress of the fluid
$\mu_{\beta }$	plastic dynamic viscosity
$\tilde{u}$	velocity in $\tilde{x}$-direction
$\tilde{v}$	velocity in $\tilde{y}$-direction
ρ	density
$\tilde{p}$	pressure
μ	dynamic viscosity
β	Casson fluid parameter
k	Thermal conductivity
U	characteristic flow velocity
D	coefficient of mass diffusivity
J	current density
B	magnetic field
$B_{0}$	uniform magnetic field strength
ν	kinematic viscosity
ξ	Transformed x-coordinate
η	Transformed y-coordinate
Pr	Prandtl number
c	parameter defining weak/strong concentrations/turbulence
E	electric field
q	radiative heat flux
σ	electrical conductivity
$c_{p}$	specific heat constant
L	length among channel walls
T	flow pulsation period
St	Strouhal number
$D_{a}$	Darcy number
Re	Reynolds number
M	Hartmann number
μ	Dynamic viscosity
$\mu_{m}$	medium’s magnetic permeability
Rd	Radiation parameter
φ	concentration profile
θ	temperature profile
Sr	Soret number
Peh	Peclet number for heat transfer
Pem	Peclet number for mass transfer
ψ	stream function
ω	vorticity function
Sk	skin friction coefficient
Nu	Nusselt number
Sh	Sherwood number
ϵ	pulsating amplitude
Re	Reynolds number
